# Non-dipping blood pressure pattern is associated with higher risk of new-onset diabetes in hypertensive patients with obstructive sleep apnea: UROSAH data

**DOI:** 10.3389/fendo.2023.1083179

**Published:** 2023-02-16

**Authors:** Qin Luo, Nanfang Li, Qing Zhu, Xiaoguang Yao, Menghui Wang, Mulalibieke Heizhati, Xintian Cai, Junli Hu, Ayinigeer Abulimiti, Ling Yao, Xiufang Li, Lin Gan

**Affiliations:** ^1^ Hypertension Center of People’s Hospital of Xinjiang Uygur Autonomous Region, Xinjiang Hypertension Institute, National Health Committee Key Laboratory of Hypertension Clinical Research, Urumqi, China; ^2^ Key Laboratory of Xinjiang Uygur Autonomous Region, Hypertension Research Laboratory, Urumqi, China; ^3^ Xinjiang Clinical Medical Research Center for Hypertension (Cardio-Cerebrovascular) Diseases, Urumqi, China

**Keywords:** New-onset, diabetes, circadian blood pressure patterns, non-dipping, obstructive sleep apnea, hypertension

## Abstract

**Objective:**

Impairment of circadian blood pressure (BP) patterns has been associated with cardiovascular risks and events in individuals with hypertension and in general populations, which are more likely to be found in obstructive sleep apnea (OSA). The aim of this study was to investigate the association of non-dipping BP pattern with new-onset diabetes in hypertensive patients with OSA, based on Urumqi Research on Sleep Apnea and Hypertension (UROSAH) data.

**Materials and methods:**

This retrospective cohort study included 1841 hypertensive patients at least 18 years of age, who were diagnosed with OSA without baseline diabetes and had adequate ambulatory blood pressure monitoring (ABPM) data at enrollment. The exposure of interest for the present study was the circadian BP patterns, including non-dipping and dipping BP pattern, and the study outcome was defined as the time from baseline to new-onset diabetes. The associations between circadian BP patterns and new-onset diabetes were assessed using Cox proportional hazard models.

**Results:**

Among 1841 participants (mean age: 48.8 ± 10.5 years, 69.1% male), during the total follow-up of 12172 person-years with a median follow-up of 6.9 (inter quartile range: 6.0-8.0) years, 217 participants developed new-onset diabetes with an incidence rate of 17.8 per 1000 person-years. The proportion of non-dippers and dippers at enrollment in this cohort was 58.8% and 41.2%, respectively. Non-dippers were associated with higher risk of new-onset diabetes compared with dippers (full adjusted hazard ratio [HR]=1.53, 95% confidence interval [CI]: 1.14-2.06, *P*=0.005). Multiple subgroup and sensitivity analyses yielded similar results. We further explored the association of systolic and diastolic BP patterns with new-onset diabetes separately, and found that diastolic BP non-dippers were associated with higher risk of new-onset diabetes (full adjusted HR=1.54, 95% CI: 1.12-2.10, *P*=0.008), whereas for systolic BP non-dippers, the association was nonsignificant after adjusted the confounding covariates (full adjusted HR=1.35, 95% CI: 0.98-1.86, *P*=0.070).

**Conclusions:**

Non-dipping BP pattern is associated with an approximately 1.5-fold higher risk of new-onset diabetes in hypertensive patients with OSA, suggesting that non-dipping BP pattern may be an important clinical implication for the early prevention of diabetes in hypertensive patients with OSA.

## Introduction

The global number of adult patients with diabetes between 20 and 79 years reached 537 million and was responsible for 6.7 million deaths in 2021 (1). Among Chinese adults, the number of patients with diabetes was 111.6 million in 2019 and will reach 147 million in 2045 (2, 3). Early prevention for diabetes is pivotal to reduce the disease burden.

Obstructive sleep apnea (OSA), a condition characterized by intermittent hypoxia and sleep fragmentation due to a complete or partial collapse of the upper airway, is highly prevalent in parallel with the obesity epidemic trends in the general population (4), with estimated nearly one billion worldwide (5). OSA and hypertension (HTN) are highly prevalent conditions in the general population, approximately 50% of OSA patients noted to have HTN (6). Accumulating evidence has confirmed a strong association between OSA, HTN and diabetes (7–10). Shared mechanisms may involve enhanced sympathetic activity, oxidative stress, systemic inflammation, activation of the hypothalamic-pituitary-adrenal axis and alteration of circulating adipokines, induced by intermittent hypoxia and sleep fragmentation (4, 11, 12). In addition, enhanced sympathetic activity can alter circadian blood pressure (BP) rhythm resulting in a non-dipping BP pattern (11, 13), which was found to be increased by approximately 1.5 times likelihood in patients with OSA compared with non-OSA (14).

Emerging evidence has revealed that the non-dipping BP pattern is associated with adverse cardiovascular risks and events in both normal and HTN participants compared to the dipping pattern (15–18). Numerous cross-sectional studies have shown a high prevalence of non-dipping phenomenon in diabetes and even in early-stage diabetes (19–21). A recently prospective study with 21-year follow-up documented an independent association between non-dipping BP pattern and risk of new-onset diabetes in a randomly selected Finnish (n=449), originally middle-aged population with/without HTN (22). However, the longitudinal association between non-dipping pattern and the risk of developing new-onset diabetes in hypertensive patients with OSA remains unexplored.

While OSA and HTN have been substantiated to be associated with a higher risk of new-onset diabetes overall (4, 7–10, 23), non-dipping BP pattern may be a possible mechanism to accelerate the development of diabetes in hypertensive patients with OSA. Hence, we aimed to investigate the association between circadian BP patterns and new-onset diabetes based on Urumqi Research on Sleep Apnea and Hypertension (UROSAH) data.

## Materials and methods

### Study design and subjects

**Figure 1:**
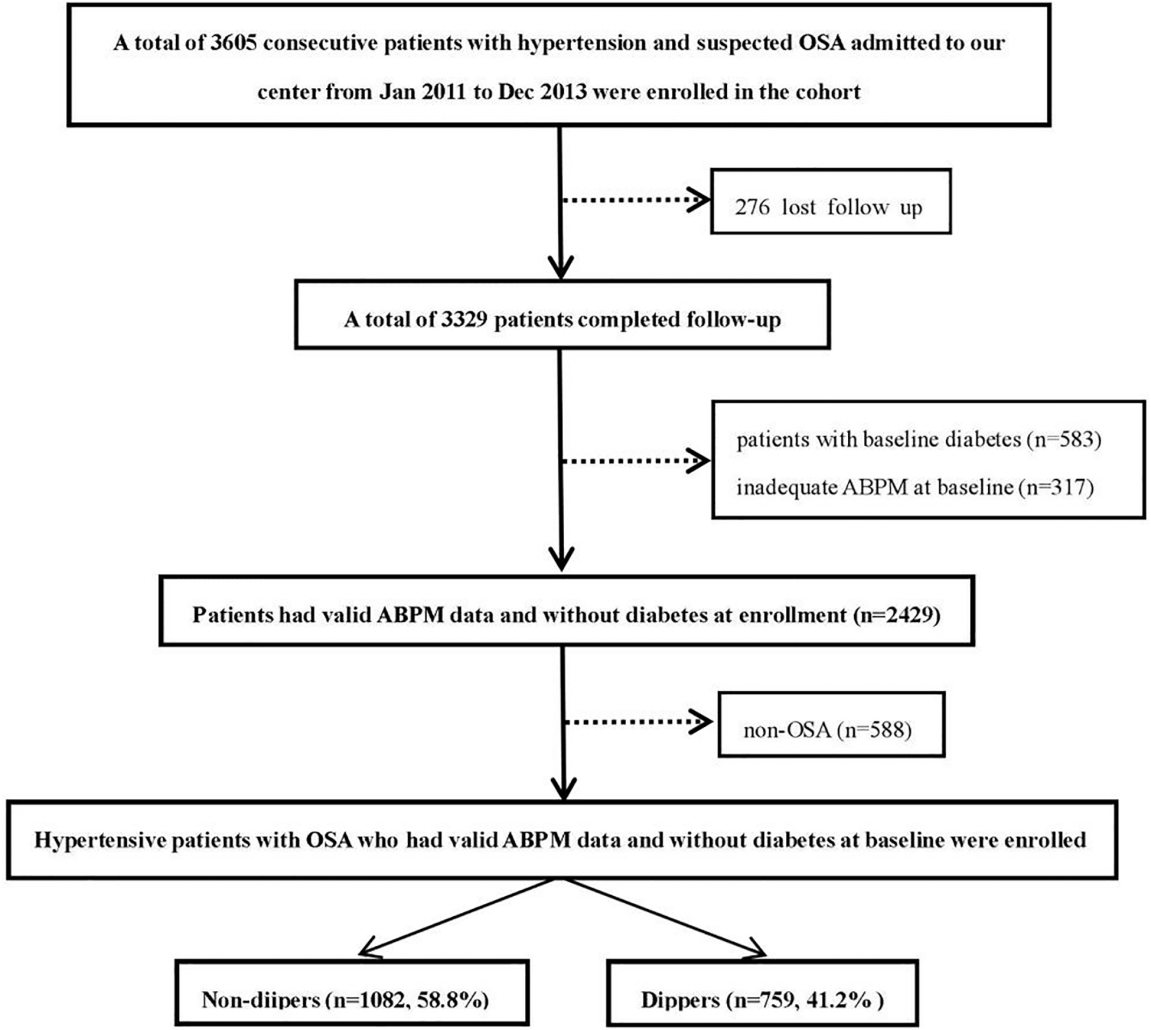
Study recruitment flowchart. OSA, obstructive sleep apnea; ABPM, ambulatory blood pressure monitoring.

Data were obtained from the UROSAH study. The design and data collection of the UROSAH study have been described in detail elsewhere (24–26). Briefly, UROSAH is a single-center observational study to assess the association of OSA with long term cardiovascular outcomes in patients with HTN. Hypertensive patients aged ≥18 years who visited Hypertension Center between Jan 2011 and Dec 2013 were reviewed. In the current study, 1841 hypertensive patients who were diagnosed with OSA without baseline diabetes and had adequate ambulatory blood pressure monitoring (ABPM) data at enrollment were included, the patient recruitment flowchart is illustrated in **Figure 1**.

### Ethical approval

The research was authorized by the Medical Ethics Committee of the People’s Hospital of Xinjiang Uygur Autonomous Region (No. 2019030662) and was conducted in strict compliance with the ethical standards set forth in the Declaration of Helsinki and its subsequent amendments. Written informed consent was submitted by all patients or their legal relatives participating in this study.

### Definitions at baseline

HTN was defined according to the “China guidelines for prevention and treatment of hypertension 2010”: the resting systolic BP (SBP) of at least 140 mmHg and/or the resting diastolic BP (DBP) of at least 90 mmHg or the current use of antihypertensive drugs (27).

Smoking and drinking status were stratified into two levels: current (current smoking/drinking or quit within the past 1 year) and never or former (non-smokers/drinkers or those who quit more than 1 year).

The height and weight were measured and body mass index (BMI) was calculated by weight (kg)/height (m^2^), obesity was defined as BMI≥28 kg/m^2^ and overweight as 24 kg/m^2^ ≤ BMI <28 kg/m^2^ according to the Criteria of Weight for Adults of the health industry standard of China, WS/T 428–2013.

Baseline prediabetes included impaired fasting glucose (IFG) and impaired glucose tolerance (IGT). IFG was defined if a fasting plasma glucose (FPG) ranged from 6.1 to less than 7.0 mmol/L, whereas 2-h glucose was less than 7.8 mmol/L; IGT was defined if 2-h glucose ranged from 7.8 to less than 11.0 mmol/L (28).

OSA was defined as an apnea-hypopnea index (AHI) ≥5 events per hour based on polysomnography (PSG) examination. Severity of OSA was defined as follows: mild OSA (5≤AHI<15 events per hour) and moderate-severe OSA (AHI≥15 events per hour) (5).

Regular continuous positive airway pressure (CPAP) treatment was defined as the use of CPAP therapy for more than 70% of nights throughout the follow-up period and no less than 4 hours per night.

### Exposure of interest

The exposure of interest was the circadian BP patterns according to the ABPM parameters. Twenty-four-hour ABPM was performed at enrollment using an oscillometric recorder (Spacelabs 90217). Briefly, the cuff with appropriate size was fitted in the non-dominant arm. After device application, subjects were encouraged to follow their usual daily activity for the next 24 h. The device was programmed to automatically measure BP every 20 min during daytime (from 8:00 to 23:00) and every 30 min during nighttime (from 23:00 to 08:00). Patients were asked to go to bed at 23:00 and not to rise before 8:00 AM. Adherence to this schedule was checked from the diary card. Only ABPM reports with more than 70% of successful readings were considered valid and included in the analysis.

Dippers were participants whose nighttime (asleep) SBP and/or DBP fall ≥10% compared with that of daytime (awake), and those with nighttime SBP and DBP fall <10% were defined as non-dippers, as indicated by the European Society of Hypertension (ESH) guidelines (29).

Elevated BP were according to the following criteria: SBP ≥130 mmHg and/or DBP ≥80 mmHg during 24-hour ABPM recording, SBP ≥135 mmHg and/or DBP ≥85 mmHg during daytime ABPM recording, SBP ≥120 mmHg and/or DBP ≥70 mmHg during nighttime ABPM recording (29).

### Follow-up and outcome

All participants were followed up through medical records, outpatient and/or inpatient visits and telephone communication. The deadline for follow-up was January 2021. All events were certified by medical documents and confirmed by the clinical event committee. Details were described in previous studies (24–26).

The outcome for the present study was defined as time from baseline to new-onset diabetes, which was determined by the WHO criteria: diabetes was diagnosed if fasting plasma glucose was ≥7.0 mmol/L and/or 2-h plasma glucose was ≥11.1 mmol/L in 2-h oral glucose tolerance test, or if a person was use of antidiabetic medications.

### Statistical analysis

The participants were divided into non-dippers and dippers according to their circadian BP patterns. Continuous variables were reported as mean ± standard deviation (SD) if normally distributed and as median and inter quartile range (IQR) if not. The differences between the two groups were compared using independent sample t-tests for normally distributed continuous variables and Mann-Whitney U-tests for non-normally distributed continuous variables. The categorical variables were presented as observed numbers and percentages and were compared among groups using Pearson’s chi-squared test.

The incidence rate of new-onset diabetes was calculated by dividing the number of incident cases by the total follow-up duration (person-years). Cumulative hazards were estimated by Kaplan-Meier curves stratified by time-updated exposure (non-dippers versus dippers) by the log-rank test. To evaluate the validity of the proportional hazard assumption, the assumption was evaluated using the log-minus-log-survival function and was found to be valid. Cox proportional hazard regression models were used to compare the risk of new-onset diabetes across groups.

**Figure 2:**
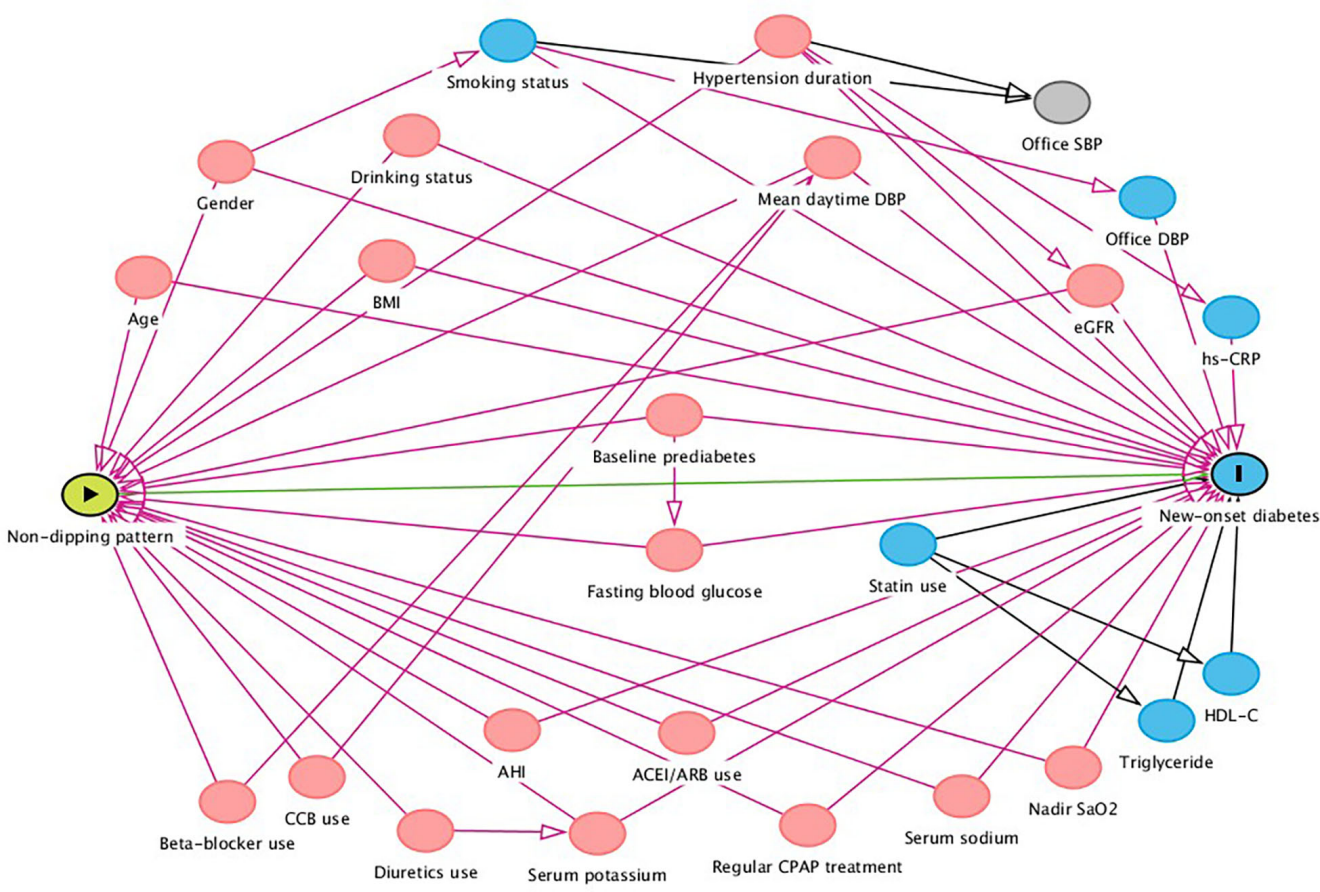
Directed acyclic graph of causal assumptions. Nodes represent variables and arrows represent causal associations. 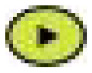
exposure 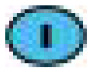
outcome 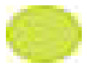
ancestor of exposure 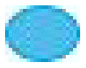
ancestor of outcome 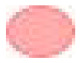
ancestor of exposure and outcome; SBP, systolic blood pressure; DBP, diastolic blood pressure; LDL-C, low-density lipoprotein cholesterol; HDL-C, high-density lipoprotein cholesterol; hs-CRP, high sensitivity C-reactive protein; eGFR, estimated glomerular filtration rate; ACEI, angiotensin-converting-enzyme inhibitor; ARB, angiotensin II receptor blocker; AHI, apnea hypopnea index; SaO_2_, oxygen saturation; CPAP, continuous positive airway pressure.

Hazard ratios (HRs) with 95% confidence intervals (CIs) were calculated, with dippers group as the reference group. Univariate Cox regression analysis was performed to select variables for adjustment (**Supplementary Table 1**). Before building the Cox regression model, we evaluated the covariance between variables according to the variance inflation factor (VIF) (**Supplementary Table 1**). Variables with VIF > 5 were considered inappropriate for inclusion in the multivariate Cox regression model. Thus, we eliminated 19 variables with multicollinearity, including waist circumference, waist-to-height ratio, serum creatinine, 24-h mean SBP, DBP, MAP and heart rate (HR), mean daytime SBP, MAP and HR, mean nighttime SBP, DBP, MAP and HR, elevated daytime BP, elevated nighttime BP, isolated elevated nighttime BP, SBP night-to-day ratios and DBP night-to-day ratios. Variables in multivariate analyses included traditional risk factors (model 1), further plus variables that gave p values <0.1 in the univariate analyses (model 2). We performed directed acyclic graphs (DAGs) by the program DAGitty for drawing and analyzed causal diagrams between non-dipping pattern and new-onset diabetes, to identify suitable minimally sufficient adjustment sets as full adjusted model (**Figure 2**, model 3).

Subgroup analyses with interaction tests were also conducted (**Supplementary Table 2**). Stratification of the study included gender (male and female), age (≥ 60 and < 60 years), BMI (≥ 28 and < 28 kg/m^2^), current drinker (yes or no), hypertension duration (≥ 5 and < 5 years), AHI (≥ 15 and < 15 events/hour), angiotensin- converting-enzyme inhibitors (ACEIs)/angiotensin II receptor blockers (ARBs) use (yes or no), and statins use (yes or no). Sensitivity analyses were performed in participants excluding baseline prediabetes, regular CPAP treatment, eGFR<60 mL/min/1.73 m^2^, statins use and normal nighttime BP (**Supplementary Table 2**). A competing risk analysis with death was performed using the Fine-Gray model (**Supplementary Table 3**). Data were analyzed using SPSS statistical software (version 25.0, SPSS Inc., Chicago, Illinois) and R (version 4.2.1) software all analyses were two-tailed and *P* value<0.05 was statistically significant.

## Results

### Baseline characteristics

Overall, the proportion of non-dippers and dippers at enrollment in the present study was 58.8% and 41.2%, respectively. The characteristics of the study population at baseline are presented in **Table 1**. Compared with dippers, non-dippers had older age, less frequent male and current drinkers. No significant differences were found in BMI, waist circumference, waist-height ratio, office SBP and DBP levels, as well as in proportion of current smokers, obesity, overweight and baseline prediabetes between non-dippers and dippers.

**Table 1:** Baseline characteristics for the total, non-dippers, and dippers.

Characteristics	Total	Non-dippers	Dippers	*P* value
Participants, n (%)	1841	1082	759	
Demographic characteristics				
Male, n (%)	1273 (69.1%)	727 (67.2%)	546 (71.9%)	0.030
Age, years	48.80 ± 10.47	49.40 ± 10.49	47.96 ± 10.40	0.004
Current smokers, n (%)	656 (35.6%)	372 (34.4%)	284 (37.4%)	0.180
Current drinkers, n (%)	644 (35.0%)	353 (32.6%)	291 (38.3%)	0.011
Body mass index, kg/m^2^	28.23 ± 3.82	28.31 ± 3.90	28.11 ± 3.71	0.253
Waist circumference, cm	100.00 (93.00, 106.00)	100.00 (93.00, 107.00)	100.00 (94.00, 106.00)	0.739
waist-to-height ratio	0.58 (0.55, 0.63)	0.58 (0.55, 0.63)	0.58 (0.55, 0.62)	0.322
Obese, n (%)	891 (48.4%)	522 (48.2%)	369 (48.6%)	0.875
Overweight, n (%)	752 (40.8%)	446 (41.2%)	306 (40.3%)	0.698
Office SBP, mmHg	139.42 ± 19.42	139.37 ± 19.52	139.50 ± 19.27	0.892
Office DBP, mmHg	92.00 ± 13.94	92.19 ± 14.01	91.72 ± 13.84	0.471
Hypertension duration, years	3.0 (1.0, 8.0)	4.0 (1.0, 8.0)	3.0 (0.8, 7.0)	0.007
Baseline prediabetes, n (%)	239 (12.9%)	146 (13.5%)	93 (12.3%)	0.436
Clinical laboratory measurements				
Total cholesterol, mmol/L	4.54 ± 1.19	4.50 ± 1.02	4.61 ± 1.23	0.033
Triglyceride, mmol/L	2.08 ± 1.50	2.02 ± 1.36	2.17 ± 1.65	0.031
HDL-C, mmol/L	1.11 ± 0.30	1.12 ± 0.31	1.10 ± 0.28	0.308
LDL-C, mmol/L	2.66 ± 0.79	2.65 ± 0.81	2.67 ± 0.76	0.587
Fasting blood glucose, mmol/L	4.85 ± 0.63	4.83 ± 0.60	4.87 ± 0.66	0.138
Serum potassium, mmol/L	3.91 ± 0.34	3.88 ± 0.34	3.96 ± 0.34	<0.001
Serum sodium, mmol/L	140.69 ± 2.38	140.79 ± 2.50	140.56 ± 2.20	0.036
hs-CRP, mg/mL (M, IQR)	1.95 (1.95, 3.62)	2.04 (0.98, 3.71)	1.87 (0.89, 3.50)	0.053
Serum creatinine, umol/L	77.07 ± 20.00	77.23 ± 21.26	76.84 ± 18.03	0.673
eGFR, mL/min/1.73 m^2^	90.18 ± 19.26	89.45 ± 18.92	91.24 ± 19.70	0.049
ABPM parameters				
Mean 24-h SBP, mmHg	133.16 ± 15.29	134.64 ± 15.56	131.05 ± 14.66	<0.001
Mean 24-h DBP, mmHg	85.61 ± 10.74	86.57 ± 11.00	84.23 ± 10.22	<0.001
24-h MAP, mmHg	100.49 ± 12.87	101.88 ± 12.98	98.51 ± 12.45	<0.001
Mean 24-h HR, bpm	75.63 ± 9.05	75.39 ± 9.27	75.96 ± 8.71	0.180
Mean daytime SBP, mmHg	136.40 ± 15.61	135.78 ± 15.75	137.29 ± 15.38	0.040
Mean daytime DBP, mmHg	88.32 ± 23.04	87.34 ± 11.11	89.72 ± 33.30	0.028
Daytime MAP, mmHg	102.68 ± 13.21	102.50 ± 13.00	102.93 ± 13.44	0.492
Mean daytime HR, bpm	79.81 ± 9.54	79.27 ± 9.63	80.60 ± 9.35	0.003
Mean nighttime SBP, mmHg	128.22 ± 16.88	133.47 ± 16.50	120.74 ± 14.43	<0.001
Mean nighttime DBP, mmHg	81.98 ± 11.65	85.57 ± 11.42	76.86 ± 9.94	<0.001
Nighttime MAP, mmHg	97.04 ± 13.79	101.14 ± 13.53	91.10 ± 11.89	<0.001
Mean nighttime HR, bpm	68.16 ± 8.89	68.48 ± 8.95	67.70 ± 8.79	0.064
Elevated 24-h BP, n (%)	1416 (76.9%)	858 (79.3%)	558 (73.5%)	0.004
Elevated daytime BP, n (%)	1272 (69.1%)	724 (66.9%)	548 (72.2%)	0.016
Elevated nighttime BP, n (%)	1649(89.6%)	1032 (95.4%)	617 (81.3%)	<0.001
Isolated elevated nighttime BP, n (%)	561 (30.5%)	416 (38.4%)	145 (19.1%)	<0.001
SBP night-to-day ratios	0.94 ± 0.07	0.98 ± 0.05	0.88 ± 0.04	<0.001
DBP night-to-day ratios	0.93 ± 0.08	0.98 ± 0.05	0.87 ± 0.05	<0.001
Prescribed medication, n (%)				
Numbers of antihypertensive drugs				
None, n(%)	167 (9.1%)	88 (8.1%)	79 (10.4)%)	0.094
1, n (%)	528 (28.7%)	288 (26.6%)	240 (31.6%)	0.019
2, n (%)	999 (54.3%)	608 (56.2%)	391 (51.5%)	0.047
3, n (%)	147 (8.0%)	98 (9.1%)	49 (6.5%)	0.043
ACEI/ARB users, n (%)	894 (48.6%)	515 (47.6%)	379 (49.9%)	0.323
Calcium channel blocker users, n (%)	1353 (73.5%)	829 (76.6%)	524 (69.0%)	<0.001
Beta blocker users, n (%)	177 (9.6%)	104 (9.6%)	73 (9.6%)	0.997
Diuretic users, n (%)	284 (15.4%)	185 (17.1%)	99 (13.0%)	0.018
Statis users, n (%)	625 (33.9%)	365(33.7%)	260(34.4%)	0.816
PSG parameters				
AHI, events/hour	19.00 (10.35, 31.25)	19.30 (10.50, 31.60)	18.60 (10.00, 30.10)	0.196
Moderate-severe OSA, n (%)	1025 (58.5%)	618 (59.8%)	407 (56.7%)	0.190
Nadir SaO_2_, %	80 (75, 84)	80 (75, 83)	80 (76, 84)	0.123
Mean SaO_2_, %	91.86 ± 3.66	91.76 ± 3.60	92.00 ± 3.73	0.183
Regular CPAP treatment, n (%)	49 (2.7%)	29 (2.7%)	20 (2.6%)	0.953

Non-normally distributed variables are expressed as the median (inter quartile range). All other values are expressed as mean ± SD or n, %.

SBP, systolic blood pressure; DBP, diastolic blood pressure; LDL-C, low-density lipoprotein cholesterol; HDL-C, high-density lipoprotein cholesterol; hs-CRP, high sensitivity C-reactive protein; IQR, inter quartile range; eGRF, estimated glomerular filtration rate; APBM, ambulatory blood pressure monitoring; MAP, mean arterial pressure; HR, heart rate; bpm, beats per minute; BP, blood pressure; ACEI, angiotensin-converting-enzyme inhibitor; ARB, angiotensin II receptor blocker; PSG, polysomnography; AHI, apnea hypopnea index; OSA, obstructive sleep apnea; SaO_2_, oxygen saturation; CPAP, continuous positive airway pressure.

As for clinical laboratory measurements, non-dippers had lower levels of total cholesterol, triglyceride (TG), serum potassium, eGFR and higher levels of serum sodium than dippers, no significant differences were found in levels of high-density lipoprotein cholesterol (HDL-C), low-density lipoprotein cholesterol (LDL-C), fasting blood glucose and serum creatinine between non-dippers and dippers.

For ABPM parameters, as expected, non-dippers had higher levels of mean 24-h and nighttime SBP, DBP and mean arterial pressure (MAP), more frequent elevated 24-h and nighttime BP, whereas lower levels of mean daytime SBP, DBP and HR, as well as less frequent elevated daytime BP than dippers.

Non-dippers received more calcium channel blockers (CCBs), diuretics and numbers of antihypertensive agents than dippers. No significant differences were found in the proportion of ACEI/ARBs and beta blockers use, as well as in major PSG parameters (e.g., AHI values, nadir SaO_2_ levels and proportion of regular CPAP treatment) between non-dippers and dippers.

### Risk of new-onset diabetes in groups by non-dippers and dippers

**Figure 3:**
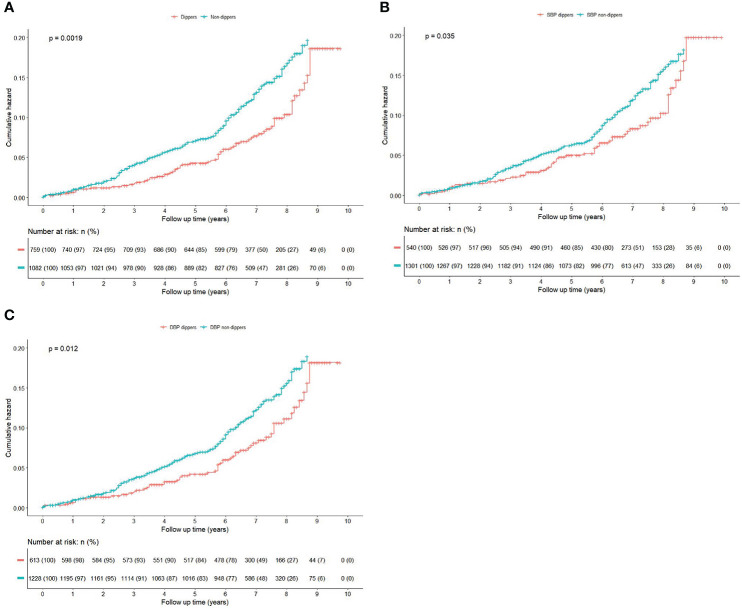
(Color online) Kaplan-Meier curves for the cumulative risk of new-onset diabetes by dipping and non-dipping pattern during the follow-up. Kaplan-Meier curves were compared with the log-rank test. **(A)** BP non-dippers vs dippers; **(B)** Systolic BP non-dippers vs dippers; **(C)** Diastolic BP non-dippers vs dippers.

Among 1841 participants (mean age: 48.8 ± 10.5 years, 69.1% male), during the total follow-up of 12172 person-years with a median follow-up of 6.9 (inter quartile range: 6.0-8.0) years, 217 participants developed new-onset diabetes with an incidence rate of 17.8 per 1000 person-years. Non-dippers experienced a higher cumulative hazard of new-onset diabetes than dippers during the follow-up period (*P*=0.0019 for log-rank test; **Figure 3A**). In univariate cox regression analysis (**Supplementary Table 1**), BMI, waist circumference, waist-to-height ratio, baseline prediabetes, TG, HDL-C, fasting blood glucose, statins use, AHI and nadir SaO_2_ were associated with higher risk of new-onset diabetes. Apart from mean daytime DBP, SBP night-to-day ratios and DBP night-to-day ratios, no other ABPM parameter showed an association with new-onset diabetes.

Three multivariate models were performed through the following sequential adjustments: model 1 adjusted for traditional risk factors, including age, gender, drinking status, smoking status, BMI, office SBP, office DBP, hypertension duration and baseline prediabetes. Model 2 included covariates in Model 1 and further adjusted for variables that gave p values <0.1 in the univariate analyses and no multicollinearity, including TG, HDL-C, fasting blood glucose, mean daytime DBP, ACEIs/ARBs use, CCBs use, statins use, AHI and nadir SaO_2_. Model 3 (full adjusted model) was based on minimal sufficient adjustment sets for estimating the total effect of non-dipping pattern on new-onset diabetes identified by DAG (**Figure 2**), including age, gender, drinking status, hypertension duration, baseline prediabetes, BMI, fasting blood glucose, eGFR, serum potassium, serum sodium, mean daytime DBP, ACEI/ARBs use, AHI, nadir SaO_2_, and regular CPAP treatment. Non-dippers showed an increased risk for new-onset diabetes compared with dippers in crude model with an unadjusted HR of 1.57 (95% CI 1.18-2.09, *P*=0.002). The association remained consistently in adjusted models (full adjusted HR=1.53, 95% CI: 1.14-2.06, *P*=0.005) (**Table 2**).

**Table 2:** Multivariate Cox regression analysis of association between circadian BP patterns and new-onset diabetes.

Variable	New-onset diabetes n (%)	Incidence rate per 1000 person-years	Crude Model		Model 1		Model 2		Model 3	
			HR (95% CI)	*P*	HR (95% CI)	*P*	HR (95% CI)	*P*	HR (95% CI)	*P*
BP patterns (SBP and/or DBP)										
Dippers (n=759)	68 (9.0)	13.4	1 [reference]		1 [reference]		1 [reference]		1 [reference]	
Non-dippers (n=1082)	149 (13.8)	21.0	1.57 (1.18-2.09)	0.002	1.41 (1.05-1.89)	0.022	1.51 (1.12-2.03)	0.006	1.53 (1.14-2.06)	0.005
SBP patterns										
Dippers (n=540)	51 (9.4)	14.0	1 [reference]		1 [reference]		1 [reference]		1 [reference]	
Non-dippers (n=1301)	166 (12.8)	19.5	1.40 (1.02-1.92)	0.036	1.25 (0.91-1.72)	0.169	1.30 (0.95-1.80)	0.106	1.35 (0.98-1.86)	0.070
DBP patterns										
Dippers (n=613)	56 (9.1)	13.7	1 [reference]		1 [reference]		1 [reference]		1 [reference]	
Non-dippers (n=1228)	161 (13.1)	19.9	1.47 (1.09-2.00)	0.013	1.35 (0.99-1.84)	0.057	1.52 (1.11-2.08)	0.009	1.54 (1.12-2.10)	0.008

Crude model: Unadjusted. Model 1: adjusted for age, gender, drinking status, smoking status, BMI, office SBP, office DBP, hypertension duration, baseline prediabetes. Model 2: adjusted for all the variables in model 1, plus triglyceride, HDL-C, fasting blood glucose, mean daytime DBP, ACEI/ARBs use, CCBs use, statins use, AHI and nadir SaO_2_. Model 3: based on minimal sufficient adjustment sets for estimating the total effect of non-dipping pattern on new-onset diabetes: age, gender, drinking status, hypertension duration, baseline prediabetes, BMI, fasting blood glucose, eGFR, serum potassium, serum sodium, mean daytime DBP, ACEI/ARBs use, AHI, nadir SaO_2_, and regular CPAP treatment.

HR, hazard ratio; BMI, body mass index; SBP, systolic blood pressure; DBP, blood pressure; HDL-C, high-density lipoprotein cholesterol; ACEI, angiotensin-converting-enzyme inhibitor; ARB, angiotensin II receptor blocker; CCB, calcium channel blocker; AHI, apnea hypopnea index; SaO_2_, oxygen saturation; hs-CRP, high sensitivity C-reactive protein; eGRF, estimated glomerular filtration rate.

We also explored association of systolic and diastolic BP patterns with new-onset diabetes separately (**Figures 3B, C**), and found that DBP non-dippers were associated with new-onset diabetes both in crude model (unadjusted HR=1.47, 95% CI:1.09-2.00, *P*=0.013) and adjusted models (full adjusted HR=1.54, 95%CI:1.12-2.10, *P*=0.008), whereas for SBP non-dippers, the association was only significantly in crude model (unadjusted HR=1.40, 95% CI: 1.02-1.92, *P*=0.036), after adjusted the aforementioned covariates, the association was nonsignificant (full adjusted HR=1.35, 95% CI: 0.98-1.86, *P*=0.070) (**Table 2**).

### Subgroup and sensitivity analysis

In subgroup analysis, as shown in **Table 3**, compared with dippers, non-dippers exhibited higher risk for new-onset diabetes in participants whom were male, age<60 years, non-obese, current drinker, hypertension duration<5 years, moderate-severe OSA, no use of ACEI/ARBs and statins. None of above variables, substantially altered the association between non-dipping pattern and risk of new-onset diabetes (*P* for all interaction > 0.05).

**Table 3:** Association between non-dipping pattern and new-onset diabetes stratified by subgroups.

Subgroup	N	New-onset diabetes, n (%)	Incidence rate per 1000 person-years	Full adjusted HR (95% CI)	*P*	*P* for interaction
Gender						0.532
Male	1273	144 (11.3)	17.0	1.65 (1.15-2.36)	0.007	
Female	568	73 (12.9)	19.8	1.40 (0.83-2.37)	0.208	
Age, years						0.170
≥60	286	40 (14.0)	21.9	1.27 (0.59-2.71)	0.545	
<60	1555	177 (11.4)	17.1	1.57 (1.13-2.17)	0.007	
BMI, kg/m^2^						0.459
≥28	891	131 (14.7)	22.6	1.30 (0.89-1.91)	0.176	
<28	950	86 (9.1)	13.5	2.33 (1.42-3.83)	0.001	
Current Drinker						0.165
yes	644	67 (10.4)	15.4	1.90 (1.11-3.25)	0.019	
no	1197	150 (12.5)	19.2	1.40 (0.98-2.02)	0.065	
Hypertension duration						0.322
≥5 years	773	95 (12.3)	18.9	1.36 (0.86-2.14)	0.191	
<5 years	1068	122 (12.4)	17.1	1.81 (1.21-2.70)	0.004	
AHI, events/hour						0.637
≥15	1116	153 (13.6)	20.9	1.72 (1.19-2.47)	0.004	
<15	725	65 (9.0)	13.4	1.21 (0.71-2.05)	0.490	
ACEI/ARBs use						0.677
yes	894	120 (13.4)	20.2	1.40 (0.95-2.07)	0.094	
no	947	97 (10.2)	15.6	1.78 (1.11-2.85)	0.017	
Statins use						0.817
yes	625	94 (15.0)	23.9	1.54 (0.98-2.41)	0.059	
no	1216	123 (10.1)	14.9	1.60 (1.06-2.41)	0.024	

full adjusted model: based on minimal sufficient adjustment sets for estimating the total effect of non-dipping pattern on new-onset diabetes: age, gender, drinking status, hypertension duration, baseline prediabetes, BMI, fasting blood glucose, eGFR, serum potassium, serum sodium, mean daytime DBP, ACEI/ARBs use, AHI, nadir SaO_2_, and regular CPAP treatment.

BMI, body mass index; eGRF, estimated glomerular filtration rate; AHI, apnea hypopnea index; SaO_2_, oxygen saturation; ACEI, angiotensin-converting-enzyme inhibitor; ARB, angiotensin II receptor blocker.

Sensitivity analysis showed that the association of non-dipping BP pattern with the risk of new-onset diabetes didn’t change in participants excluding baseline prediabetes, regular CPAP treatment, eGFR<60 mL/min/1.73 m^2^, statins use and normal nighttime BP (**Supplementary Table 2**). We also performed a competing risk analysis with death as a competing event. Sub-distribution HRs for non-dipping pattern and new-onset diabetes after competing risk analysis are presented in **Supplementary Table 3**. The association between non-dipping pattern and new-onset diabetes remained significant in the Fine Gray model.

## Discussion

The current study is, to the best of our knowledge, the first to explore the association of nocturnal BP patterns with new-onset diabetes in a relatively large cohort of hypertensive patients with OSA. Our findings suggest that non-dippers, despite having similar levels of office SBP, DBP, and even lower mean daytime SBP and DBP compared to dippers, are associated with a higher risk of new-onset diabetes in hypertensive patients with OSA.

ABPM can assess circadian BP patterns over a 24-hour period and provides more useful prognostic information than clinic measurements of BP. The proportion of non-dippers at enrollment in the current cohort was as high as 58.8%, which is consistent with previous studies. A meta-analysis involving 1562 patients with OSA and 957 non-OSA controls from 14 studies revealed that the prevalence of non-dipping patterns in patients with OSA varied widely from 36.0% to 90.0% and non-OSA from 33.0% to 69.0%, depending on demographic or clinical characteristics, definitions of OSA and non-dipping phenotypes (14).

The dipping phenomenon occurs when lying recumbent due to lower leg fluid shifts in the rostral direction, increasing carotid intravascular fluid volume and triggering carotid baroreceptors to reflexively reduce sympathetic nervous activity (SNA), thus causing a nocturnal BP dipping. The enhanced SNA caused by OSA can antagonize the natural dipping phenomenon, meanwhile, the chronic HTN leading to endothelial dysfunction, vasculature abnormity, and insensitive baroreceptors may further inhibit the reflex dipping phenomenon (30).

In animal models as well as in humans, exposure to intermittent hypoxia and disruption of circadian rhythms have been shown to be associated with pancreatic beta cell loss and dysfunction, metabolic abnormalities, impaired function of the autonomic nervous system, renin-angiotensin system, and organ malfunction in the target organ (31, 32). Insulin resistance may in turn contribute to the development of non-dipper hypertension (33). The above superimposed changes may further aggravate metabolic abnormalities and associated cardiovascular events.

Previous studies paid more attention to SBP patterns than to DBP (34). In the present study, we explored the association of both systolic and diastolic BP patterns with new-onset diabetes and found that DBP non-dippers were significantly associated with higher risk of new-onset diabetes after adjustment for confounding factors, while SBP non-dippers were non-significant after full adjustment. Our findings are in agreement with several studies that have focused on the association of both SBP and DBP modes with OSA. A case-control study showed that patients with OSA had increased ambulatory DBP during both day and night and increased SBP during the night, compared to closely matched control subjects (35). A retrospective study found that subjects with more severe intermittent hypoxia and sleep fragmentation had significant higher SBP and DBP, and were more likely to have abnormal DBP than those with less severe intermittent hypoxia and sleep fragmentation (36). These findings suggest that DBP mode is more likely to be specific in patients with OSA. A longitudinal analysis of the Wisconsin Sleep Cohort indicated that there was a dose-response greater risk of developing both SBP and DBP non-dipping patterns with greater severity of OSA in rapid eye movement (REM) sleep (37). Moreover, DBP non-dipping was significantly associated with the REM AHI, but not non-REM or total AHI (37), while SBP non-dipping was significantly associated with total AHI (30). It is well established that REM sleep is associated with greater sympathetic activity and cardiovascular instability in patients with OSA, which may explain why the risk of new onset diabetes subtly differs between SBP and DBP non-dipping patterns.

Elevated sleep-time BP has also been proposed as an important prognostic marker of diabetes. MAPEC study comprising 2,656 individuals without diabetes and with baseline BP ranging from normotension to HTN, during a 5.9-year follow-up, indicated that elevated sleep-time SBP is an independent prognostic marker for new-onset diabetes and lowering asleep BP could be a significant method for reducing new-onset diabetes risk. Moreover, those with new-onset diabetes were likely to have OSA at baseline (38). Nonetheless, neither mean nighttime SBP nor DBP was found to be associated with new-onset diabetes in the present study, the main reason for this may be attributed to the relatively high percentage of participants with elevated nighttime BP, 89.6% for the whole cohort, 95.4% for the non-dippers and 81.3% for the dippers, and 90.9% of the participants were on antihypertensive treatment. In the sensitivity analysis, the association between non-dippers and the risk of new-onset diabetes was unchanged in those excluding normal nighttime BP.

Currently, solid evidences of adverse prognosis of non-dipping pattern on cardiovascular risks and events provide justification for complete 24-h BP control as the primary goal of antihypertensive treatment. We therefore support the role of ABPM as an inexpensive, widely available screening and monitoring tool for the diagnosis of abnormal BP patterns in hypertensive patients with OSA, pursuing an optimized treatment and management of non-dipping BP for preventing diabetes.

CPAP is the current standard of treatment for OSA and seems to improve nocturnal BP dipping (39). Meta-analyses have shown a significant decrease in nighttime BP as well as a long-term (12 weeks) reduction in mean and diastolic BP with CPAP device usage of 4 or more hours per night (40, 41). A meta-analyses included six randomized controlled trials revealed that CPAP therapy has a favorable effect on insulin resistance in adult participants with OSA without diabetes (42). VAMONOS study demonstrated that only an outstanding compliance (defined as ≥90% of nights and 8 h/night) to CPAP reduced fasting blood glucose in patients with OSA. Longitudinally, higher levels of therapeutic adherence may affect the rate of incident impaired fasting glucose, prediabetes, and type 2 diabetes mellitus (T2DM), despite the observed weight gains (43). A recent meta-analysis included seven trials (enrolling 691 participants) determined that CPAP treatment significantly improved glycemic control and insulin resistance in patients with T2DM and contemporary OSA (44). However, CPAP therapy remained so far mixed results in improving glucose metabolism (45), herein, non-dipping BP pattern could be an important hallmark to determine high-risk patients and may need to be considered as an important clinical implication for early prevention of diabetes in hypertensive patients with OSA.

BP lowering has been an established strategy for the prevention of new-onset type 2 diabetes in hypertensive patients (46). To achieve 24-h BP control, evening or bedtime administration of antihypertensive drugs has been proposed as a potentially more effective strategy to control nocturnal hypertension, normalize the night-time BP dip. Previous study indicated that bedtime HTN treatment, in conjunction with proper patient evaluation by ABPM to corroborate the diagnosis of HTN and avoid treatment-induced nocturnal hypotension, should be the preferred therapeutic scheme for type 2 diabetes mellitus. However, it might be argued that bedtime dosing is a reasonable approach to be applied specifically to non-dippers, or patients with isolated night-time hypertension. To date, the supporting evidence, clinical relevance and indications for bedtime dosing remain debatable. The current evidence on the comparative impact of bedtime versus other times of antihypertensive drug dosing on 24-h BP profile and on cardiovascular morbidity and mortality is limited by insufficient design and/or rigor of the available studies. The relevant ongoing trails and their results are expected to shed light on the impact of bedtime versus morning drug dosing on outcome (47). Treatment In Morning versus Evening (TIME) study has recently revealed that evening dosing of commonly used anti-hypertensive medications is no different from morning dosing in terms of major cardiovascular outcomes in hypertensive subjects (48). However, it remains unclear which treatment (e.g., CPAP, bedtime HTN therapy or combination) is optimal for preventing diabetes in patients with non-dipping HTN and OSA, further studies may need to focus on this population (47).

Our study has some limitations. First, current guidelines recommend the assessment of average daytime and nighttime BP values to be based on the individuals’ sleeping times and the diagnosis of non-dippers to be confirmed with repeat ABPM. In this study, nighttime BP values were fixed from 23:00 to 08:00 and BP patterns were assessed only at the enrolment visit without repetition. Nonetheless, it was reported that the use of fixed-time periods may be a reasonable alternative approach for self-report in ABPM (49). Second, we failed to follow up ABPM so that the change of BP pattern was unclear, however, in a prospective study, of all dippers, only 42.7% remained dippers, while of all non-dippers, 81.4% remained non-dippers in the follow-up (22). Therefore, it is unlikely to overestimate the risk of non-dipping BP pattern for new-onset diabetes in this study. Third, only 2.7% patients received regular CPAP treatment possibly due to poor compliance or acceptance, thus we could not evaluate the effect of CPAP treatment on prevention for diabetes. In addition, we failed to record the administration time of antihypertensive drugs for each patient, nonetheless, bedtime dosing was not common in the routine prescription in our clinical practice. Fourth, this study is a retrospective cohort analysis and therefore is susceptible to residual confounding biases. Finally, UROSAH cohort is constituted by Chinese population from a single tertiary center, the results may not be extrapolated to other ethnicities, as previous studies reported that nocturnal dipping pattern differed by ethnicities (50).

## Conclusions

We demonstrated that non-dippers are associated with approximately 1.5-fold higher risk for new-onset diabetes than dippers among hypertensive patients with OSA, suggesting that non-dipping BP pattern may need to be considered as an important clinical implication for early prevention of diabetes among this population.

## Data availability statement

The raw data supporting the conclusions of this article will be made available by the authors, without undue reservation.

## Ethics statement

The studies involving human participants were reviewed and approved by Ethics Committee of the People’s Hospital of Xinjiang Uygur Autonomous Region. The patients/participants provided their written informed consent to participate in this study.

## Author contributions

QL was responsible for design, data collection, analysis and writing manuscript. NL was responsible for design, conduct, data collection and guiding the whole study. QZ, XY, MW, MH, XC, JH and LG participated in conduct/data collection and analysis. AA, LY, and XL participated in conduct/data collection. All authors contributed to the article and approved the submitted version.

## References

[1] International Diabetes Federation. IDF diabetes atlas. 10th edn. Brussels, Belgium (2021). Available at: https://www.diabetesatlas.org.

[2] Zhang F Ji L Hong T Guo L Li Y Zhu Z. Expert consensus on personalized initiation of glucose-lowering therapy in adults with newly diagnosed type 2 diabetes without clinical cardiovascular disease or chronic kidney disease. J Evidence-Based Med (2022) 15(2):168–79. doi: 10.1111/jebm.12474 35715995

[3] James SL Abate D Abate KH Abay SM Abbafati C Abbasi N. Global, regional, and national incidence, prevalence, and years lived with disability for 354 diseases and injuries for 195 countries and territories, 1990–2017: a systematic analysis for the global burden of disease study 2017. Lancet (2018) 392(10159):1789–858. doi: 10.1016/S0140-6736(18)32279-7 PMC6227754 30496104

[4] Reutrakul S Mokhlesi B. Obstructive sleep apnea and diabetes: A state of the art review. Chest (2017) 152(5):1070–86. doi: 10.1016/j.chest.2017.05.009 PMC5812754 28527878

[5] Chang JL Goldberg AN Alt JA Ashbrook L Auckley D Ayappa I. International consensus statement on obstructive sleep apnea. Int Forum Allergy Rhinol (2022). doi: 10.1002/alr.23079 PMC10359192 36068685

[6] Drager LF Genta PR Pedrosa RP Nerbass FB Gonzaga CC Krieger EM. Characteristics and predictors of obstructive sleep apnea in patients with systemic hypertension. Am J Cardiol (2010) 105(8):1135–9. doi: 10.1016/j.amjcard.2009.12.017 20381666

[7] Aurora RN Punjabi NM. Obstructive sleep apnoea and type 2 diabetes mellitus: A bidirectional association. Lancet Respir Med (2013) 1(4):329–38. doi: 10.1016/S2213-2600(13)70039-0 24429158

[8] Vacelet L Hupin D Pichot V Celle S Court-Fortune I Thomas T. Insulin resistance and type 2 diabetes in asymptomatic obstructive sleep apnea: Results of the PROOF cohort study after 7 years of follow-up. Front Physiol (2021) 12:650758. doi: 10.3389/fphys.2021.650758 34393806 PMC8355896

[9] Qie R Zhang D Liu L Ren Y Zhao Y Liu D. Obstructive sleep apnea and risk of type 2 diabetes mellitus: A systematic review and dose-response meta-analysis of cohort studies. J Diabetes (2020) 12(6):455–64. doi: 10.1111/1753-0407.13017 31872550

[10] Tsimihodimos V Gonzalez-Villalpando C Meigs JB Ferrannini E. Hypertension and diabetes mellitus: Coprediction and time trajectories. Hypertens (2018) 71(3):422–8. doi: 10.1161/HYPERTENSIONAHA.117.10546 PMC5877818 29335249

[11] Abboud F Kumar R. Obstructive sleep apnea and insight into mechanisms of sympathetic overactivity. J Clin Invest (2014) 124(4):1454–7. doi: 10.1172/JCI70420 PMC3973099 24691480

[12] Kohler M Stradling JR. CrossTalk proposal: Most of the cardiovascular consequences of OSA are due to increased sympathetic activity. J Physiol (2012) 590(12):2813–5; discussion 23. doi: 10.1113/jphysiol.2012.229633 22707583 PMC3448139

[13] Sherwood A Steffen PR Blumenthal JA Kuhn C Hinderliter AL. Nighttime blood pressure dipping: The role of the sympathetic nervous system. Am J Hypertens (2002) 15(2 Pt 1):111–8. doi: 10.1016/S0895-7061(01)02251-8 11863245

[14] Cuspidi C Tadic M Sala C Gherbesi E Grassi G Mancia G. Blood pressure non-dipping and obstructive sleep apnea syndrome: A meta-analysis. J Clin Med (2019) 8(9):1367. doi: 10.3390/jcm8091367 31480717 PMC6780266

[15] Palatini P Reboldi G Saladini F Angeli F Mos L Rattazzi M. Dipping pattern and short-term blood pressure variability are stronger predictors of cardiovascular events than average 24-h blood pressure in young hypertensive subjects. Eur J Prev Cardiol (2022) 29(10):1377–86. doi: 10.1093/eurjpc/zwac020 35104844

[16] Saeed S Waje-Andreassen U Lonnebakken MT Fromm A Oygarden H Naess H. Covariates of non-dipping and elevated night-time blood pressure in ischemic stroke patients: the Norwegian stroke in the young study. Blood Press (2016) 25(4):212–8. doi: 10.3109/08037051.2015.1127559 26694634

[17] Hjortkjaer HO Persson F Theilade S Winther SA Tofte N Ahluwalia TS. Non-dipping and higher nocturnal blood pressure are associated with risk of mortality and development of kidney disease in type 1 diabetes. J Diabetes Complicat (2022) 36(9):108270. doi: 10.1016/j.jdiacomp.2022.108270 35964524

[18] Cuspidi C Tadic M Sala C Carugo S Mancia G Grassi G. Reverse dipping and subclinical cardiac organ damage: A meta-analysis of echocardiographic studies. J Hypertens (2021) 39(8):1505–12. doi: 10.1097/HJH.0000000000002836 33657585

[19] Bromfield SG Shimbo D Bertoni AG Sims M Carson AP Muntner P. Ambulatory blood pressure monitoring phenotypes among individuals with and without diabetes taking antihypertensive medication: The Jackson heart study. J Hum Hypertens (2016) 30(12):731–6. doi: 10.1038/jhh.2016.27 PMC5338609 27169827

[20] Nikolaidou B Anyfanti P Gavriilaki E Lazaridis A Triantafyllou A Zarifis H. Non-dipping pattern in early-stage diabetes: Association with glycemic profile and hemodynamic parameters. J Hum Hypertens (2022) 36(9):805–10. doi: 10.1038/s41371-021-00587-4 34400769

[21] Aung AT Chan SP Kyaing TT Lee CH. Diabetes mellitus is associated with high sleep-time systolic blood pressure and non-dipping pattern. Postgrad Med (2020) 132(4):346–51. doi: 10.1080/00325481.2020.1745537 32208051

[22] Lempiainen PA Vasunta RL Bloigu R Kesaniemi YA Ukkola OH. Non-dipping blood pressure pattern and new-onset diabetes in a 21-year follow-up. Blood Press (2019) 28(5):300–8. doi: 10.1080/08037051.2019.1615369 31092019

[23] Yildiz M Esenboga K Oktay AA. Hypertension and diabetes mellitus: Highlights of a complex relationship. Curr Opin Cardiol (2020) 35(4):397–404. doi: 10.1097/HCO.0000000000000748 32371623

[24] Cai X Li N Hu J Wen W Yao X Zhu Q. Nonlinear relationship between Chinese visceral adiposity index and new-onset myocardial infarction in patients with hypertension and obstructive sleep apnoea: Insights from a cohort study. J Inflammation Res (2022) 15:687–700. doi: 10.2147/JIR.S351238 PMC8819537 35140499

[25] Gan L Li N Heizati M Lin M Zhu Q Hong J. Diurnal cortisol features with cardiovascular disease in hypertensive patients: A cohort study. Eur J Endocrinol (2022) 187(5):629–36. doi: 10.1530/EJE-22-0412 36070421

[26] Gan L Li N Heizhati M Lin M Zhu Q Yao X. Higher plasma aldosterone is associated with increased risk of cardiovascular events in hypertensive patients with suspected OSA: UROSAH data. Front Endocrinol (Lausanne) (2022) 13:1017177. doi: 10.3389/fendo.2022.1017177 36277704 PMC9585258

[27] Liu LS. 2010 Chinese guidelines for the management of hypertension. Zhonghua Xin Xue Guan Bing Za Zhi (2011) 39(7):579–615. 22088239

[28] Cosentino F Grant PJ Aboyans V Bailey CJ Ceriello A Delgado V. 2019 ESC Guidelines on diabetes, pre-diabetes, and cardiovascular diseases developed in collaboration with the EASD. Eur Heart J (2020) 41(2):255–323. doi: 10.1093/eurheartj/ehz486 31497854

[29] Stergiou GS Palatini P Parati G O’Brien E Januszewicz A Lurbe E. 2021 European Society of hypertension practice guidelines for office and out-of-office blood pressure measurement. J Hypertens (2021) 39(7):1293–302. doi: 10.1097/HJH.0000000000002843 33710173

[30] Hla KM Young T Finn L Peppard PE Szklo-Coxe M Stubbs M. Longitudinal association of sleep-disordered breathing and nondipping of nocturnal blood pressure in the Wisconsin sleep cohort study. Sleep (2008) 31(6):795–800. doi: 10.1093/sleep/31.6.795 18548823 PMC2442417

[31] Gale JE Cox HI Qian J Block GD Colwell CS Matveyenko AV. Disruption of circadian rhythms accelerates development of diabetes through pancreatic beta-cell loss and dysfunction. J Biol Rhythms (2011) 26(5):423–33. doi: 10.1177/0748730411416341 PMC3359760 21921296

[32] Rahman A Hasan AU Nishiyama A Kobori H. Altered circadian timing system-mediated non-dipping pattern of blood pressure and associated cardiovascular disorders in metabolic and kidney diseases. Int J Mol Sci (2018) 19(2):400. doi: 10.3390/ijms19020400 29385702 PMC5855622

[33] Anan F Takahashi N Ooie T Yufu K Saikawa T Yoshimatsu H. Role of insulin resistance in nondipper essential hypertensive patients. Hypertens Res (2003) 26(9):669–76. doi: 10.1291/hypres.26.669 14620920

[34] O’Rourke MF Safar ME Adji A. Resistant hypertension and central aortic pressure. J Hypertens (2014) 32(3):699. doi: 10.1097/HJH.0000000000000088 24477098

[35] Davies CW Crosby JH Mullins RL Barbour C Davies RJ Stradling JR. Case-control study of 24 hour ambulatory blood pressure in patients with obstructive sleep apnoea and normal matched control subjects. Thorax (2000) 55(9):736–40. doi: 10.1136/thorax.55.9.736 PMC1745863 10950890

[36] Xia Y You K Xiong Y. Relationships between cardinal features of obstructive sleep apnea and blood pressure: A retrospective study. Front Psychiatry (2022) 13:846275. doi: 10.3389/fpsyt.2022.846275 35463518 PMC9027567

[37] Mokhlesi B Hagen EW Finn LA Hla KM Carter JR Peppard PE. Obstructive sleep apnoea during REM sleep and incident non-dipping of nocturnal blood pressure: A longitudinal analysis of the Wisconsin sleep cohort. Thorax (2015) 70(11):1062–9. doi: 10.1136/thoraxjnl-2015-207231 PMC7888359 26307037

[38] Hermida RC Ayala DE Mojon A Fernandez JR. Sleep-time BP: prognostic marker of type 2 diabetes and therapeutic target for prevention. Diabetologia (2016) 59(2):244–54. doi: 10.1007/s00125-015-3748-8 26399403

[39] Bischof F Egresits J Schulz R Randerath WJ Galetke W Budweiser S. Effects of continuous positive airway pressure therapy on daytime and nighttime arterial blood pressure in patients with severe obstructive sleep apnea and endothelial dysfunction. Sleep Breath (2020) 24(3):941–51. doi: 10.1007/s11325-019-01926-z 31463779

[40] Liu L Cao Q Guo Z Dai Q. Continuous positive airway pressure in patients with obstructive sleep apnea and resistant hypertension: A meta-analysis of randomized controlled trials. J Clin Hypertens (Greenwich) (2016) 18(2):153–8. doi: 10.1111/jch.12639 PMC8031627 26278919

[41] Iftikhar IH Valentine CW Bittencourt LR Cohen DL Fedson AC Gíslason T. Effects of continuous positive airway pressure on blood pressure in patients with resistant hypertension and obstructive sleep apnea: A meta-analysis. J Hypertens (2014) 32(12):2341–50; discussion 50. doi: 10.1097/HJH.0000000000000372 25243523 PMC4291165

[42] Iftikhar IH Hoyos CM Phillips CL Magalang UJ. Meta-analyses of the association of sleep apnea with insulin resistance, and the effects of CPAP on HOMA-IR, adiponectin, and visceral adipose fat. J Clin Sleep Med (2015) 11(4):475–85. doi: 10.5664/jcsm.4610 PMC4365462 25700870

[43] Ioachimescu OC Anthony J Jr. Constantin T Ciavatta MM McCarver K Sweeney ME. VAMONOS (Veterans affairs’ metabolism, obstructed and non-obstructed sleep) study: Effects of CPAP therapy on glucose metabolism in patients with obstructive sleep apnea. J Clin Sleep Med (2017) 13(3):455–66. doi: 10.5664/jcsm.6502 PMC5337593 28095965

[44] Shang W Zhang Y Wang G Han D. Benefits of continuous positive airway pressure on glycaemic control and insulin resistance in patients with type 2 diabetes and obstructive sleep apnoea: A meta-analysis. Diabetes Obes Metab (2021) 23(2):540–8. doi: 10.1111/dom.14247 33146450

[45] Schlatzer C Schwarz EI Kohler M. The effect of continuous positive airway pressure on metabolic variables in patients with obstructive sleep apnoea. Chron Respir Dis (2014) 11(1):41–52. doi: 10.1177/1479972313516882 24431410

[46] Nazarzadeh M Bidel Z Canoy D Copland E Wamil M Majert J. Blood pressure lowering and risk of new-onset type 2 diabetes: An individual participant data meta-analysis. Lancet (2021) 398(10313):1803–10. doi: 10.1016/S0140-6736(21)01920-6 PMC8585669 34774144

[47] Stergiou G Brunstrom M MacDonald T Kyriakoulis KG Bursztyn M Khan N. Bedtime dosing of antihypertensive medications: Systematic review and consensus statement: International society of hypertension position paper endorsed by world hypertension league and European society of hypertension. J Hypertens (2022) 40(10):1847–58. doi: 10.1097/HJH.0000000000003240 35983870

[48] Mackenzie IS Rogers A Poulter NR Williams B Brown MJ Webb DJ. Cardiovascular outcomes in adults with hypertension with evening versus morning dosing of usual antihypertensives in the UK (TIME study): a prospective, randomised, open-label, blinded-endpoint clinical trial. Lancet (2022) 400(10361):1417–25. doi: 10.1016/S0140-6736(22)01786-X PMC9631239 36240838

[49] Booth JN 3rd Muntner P Abdalla M Diaz KM Viera AJ Reynolds K. Differences in night-time and daytime ambulatory blood pressure when diurnal periods are defined by self-report, fixed-times, and actigraphy: Improving the detection of hypertension study. J Hypertens (2016) 34(2):235–43. doi: 10.1097/HJH.0000000000000791 PMC4787534 26867054

[50] Profant J Dimsdale JE. Race and diurnal blood pressure patterns. A Rev meta-analysis Hypertens (1999) 33(5):1099–104. doi: 10.1161/01.HYP.33.5.1099 10334794

