# Supplementary material for: Non-dipping blood pressure pattern is associated with higher risk of new-onset diabetes in hypertensive patients with obstructive sleep apnea: UROSAH data
Source: Front Endocrinol (Lausanne). 2023 Feb 16;14:1083179. doi: 10.3389/fendo.2023.1083179 (PMC9978411; doi:10.3389/fendo.2023.1083179)
Supplement: Supplementary file 1 [file Table_1.docx]

| **Supplementary Table 1** Univariate Cox regression analysis of association between covariate factors and new-onset diabetes | | | | |
| --- | --- | --- | --- | --- |
|  | HR (95% CI) | *P* value | Tolerance | VIF |
| Traditional risk factors |  |  |  |  |
| gender (male vs female) | 0.89 (0.66-1.15) | 0.326 | 0.262 | 3.82 |
| Age | 1.01 (1.00-1.02) | 0.073 | 0.418 | 2.39 |
| Current smoker (yes vs no) | 1.08 (0.82-1.42) | 0.586 | 0.671 | 1.49 |
| Current drinker (yes vs no) | 0.78 (0.59-1.05) | 0.097 | 0.675 | 1.48 |
| Body mass index | 1.07(1.04-1.11) | <0.001 | 0.296 | 3.38 |
| Waist circumference | 1.03 (1.01-1.04) | <0.001 | 0.092 | 10.84 |
| Waist-to-height ratio (per 0.1) | 1.69 (1.38, 2.06) | <0.001 | 0.089 | 11.23 |
| Office SBP | 1.00 (0.99-1.01) | 0.887 | 0.354 | 2.82 |
| Office DBP | 0.99 (0.98-1.00) | 0.104 | 0.335 | 2.98 |
| Hypertension duration | 1.11 (0.99-1.03) | 0.105 | 0.772 | 1.30 |
| Prediabetes at baseline (yes vs no) | 3.86 (2.91-5.12) | <0.001 | 0.902 | 1.11 |
| Clinical laboratory measurements |  |  |  |  |
| Serum creatinine | 1.00 (0.99-1.00) | 0.226 | 0.195 | 5.15 |
| Total cholesterol | 0.94 (0.82-1.08) | 0.391 | 0.467 | 2.14 |
| Triglyceride | 1.09 (1.03-1.15) | 0.005 | 0.691 | 1.45 |
| HDL-C | 0.51 (0.31-0.82) | 0.006 | 0.718 | 1.39 |
| LDL-C | 0.90 (0.76-1.07) | 0.233 | 0.532 | 1.88 |
| Fasting plasma glucose | 2.73 (2.21-3.37) | <0.001 | 0.888 | 1.13 |
| Serum potassium | 1.07 (0.73-1.58) | 0.723 | 0.875 | 1.14 |
| Serum sodium | 0.99 (0.94-1.05) | 0.809 | 0.943 | 1.06 |
| hs-CRP | 1.01 (0.99-1.02) | 0.361 | 0.955 | 1.05 |
| eGFR | 1.00 (0.99-1.01) | 0.715 | 0.238 | 4.20 |
| AMBP parameters |  |  |  |  |
| 24-h mean SBP | 1.00 (0.99-1.01) | 0.662 | 0.010 | 98.03 |
| 24-h mean DBP | 0.99 (0.98-1.00) | 0.108 | 0.010 | 101.65 |
| 24-h MAP | 0.99 (0.99-1.01) | 0.347 | 0.015 | 68.94 |
| 24h mean HR | 1.01 (0.99-1.02) | 0.256 | 0.023 | 44.17 |
| Mean daytime SBP | 1.00 (0.99-1.01) | 0.870 | 0.010 | 205.55 |
| Mean daytime DBP | 0.99 (0.98-1.00) | 0.027 | 0.251 | 3.99 |
| Daytime MAP | 0.99 (0.98-1.00) | 0.135 | 0.042 | 23.93 |
| Mean daytime HR | 1.01 (0.99-1.02) | 0.288 | 0.044 | 22.94 |
| Mean nighttime SBP | 1.01 (1.00-1.01) | 0.147 | 0.003 | 328.98 |
| Mean nighttime DBP | 1.00 (0.99-1.01) | 0.594 | 0.007 | 146.28 |
| Nighttime MAP | 1.00 (0.99-1.01) | 0.947 | 0.015 | 66.78 |
| Mean nighttime HR | 1.01 (1.00-1.03) | 0.175 | 0.108 | 9.23 |
| SBP night-to-day ratios | 11.07 (1.93-63.75) | 0.007 | 0.010 | 96.75 |
| DBP night-to-day ratios | 8.39 (1.56-45.07) | 0.013 | 0.037 | 26.79 |
| Elevated 24-h BP | 1.09 (0.79-1.52) | 0.596 | 0.369 | 2.71 |
| Elevated daytime BP | 0.81 (0.61-1.08) | 0.147 | 0.032 | 31.44 |
| Elevated nighttime BP | 1.34（0.83-2.17） | 0.239 | 0.080 | 12.55 |
| Isolated elevated nighttime BP | 1.04（0.77-1.39） | 0.809 | 0.038 | 26.52 |
| Prescribed medication (yes vs no) |  |  |  |  |
| ACEI/ARBs use | 1.29 (0.99-1.69) | 0.061 | 0.903 | 1.11 |
| CCBs use | 1.37 (1.0-1.89) | 0.051 | 0.836 | 1.20 |
| Beta blockers use | 0.74 (0.43-1.26) | 0.265 | 0.907 | 1.10 |
| Diuretics use | 1.20 (0.84-1.71) | 0.322 | 0.879 | 1.14 |
| Statins use | 1.66 (1.27-2.17) | <0.001 | 0.947 | 1.06 |
| [PSG parameters](javascript:;) |  |  |  |  |
| AHI | 1.01(1.00-1.02) | 0.009 | 0.628 | 1.59 |
| Nadir SaO_2_ | 0.98 (0.96-1.00) | 0.029 | 0.668 | 1.50 |
| Mean SaO_2_ | 0.99 (0.96-1.02) | 0.496 | 0.684 | 1.46 |
| Regular CPAP treatment (yes vs no) | 1.23 (0.58-2.60) | 0.598 | 0.967 | 1.03 |
| **Abbreviations:** HR, hazard ratio; SBP, systolic blood pressure; DBP, diastolic blood pressure; LDL-C, low-density lipoprotein cholesterol; HDL-C, high-density lipoprotein cholesterol; hs-CRP, high sensitivity C-reactive protein; IQR, interquartile range; eGFR, estimated glomerular filtration rate; APBM, ambulatory blood pressure monitoring; MAP, mean arterial pressure; HR, heart rate; BP, blood pressure; ACEI, angiotensin- converting-enzyme inhibitors; ARB, angiotensin II receptor blocker; PSG, polysomnography; AHI, apnea hypopnea index; OSA, obstructive sleep apnea; Nadir SaO_2_, nadir oxygen saturation; Mean SaO_2_, mean oxygen saturation; CPAP, continuous positive airway pressure. | | | | |
