# Supplementary material for: Non-dipping blood pressure pattern is associated with higher risk of new-onset diabetes in hypertensive patients with obstructive sleep apnea: UROSAH data
Source: Front Endocrinol (Lausanne). 2023 Feb 16;14:1083179. doi: 10.3389/fendo.2023.1083179 (PMC9978411; doi:10.3389/fendo.2023.1083179)
Supplement: Supplementary file 2 [file Table_2.docx]

| **Supplementary Table 2** [Sensitivity analysis](javascript:;) of association of non-dipping pattern with new-onset diabetes in different conditions | | | | | | | |
| --- | --- | --- | --- | --- | --- | --- | --- |
| Participants with different conditions (non-dippers verse dippers) | n | New-onset diabetes  n (%) | Incidence rate per 1000  person-years | Crude Model | | Full adjusted model | |
|  |  |  |  | HR (95% CI) | *P* value | HR (95% CI) | *P* value |
| Participants without baseline prediabetes | 1602 | 143 (8.9) | 13.3 | 1.62 (1.14-2.31) | 0.007 | 1.62 (1.12-2.32) | 0.010 |
| Participants without regular CPAP treatment | 1792 | 210 (11.7) | 17.8 | 1.60 (1.19-2.04) | 0.002 | 1.57 (1.16-2.13) | 0.004 |
| Participants with eGFR ≥ 60  mL/min/1.73 m^2^ | 1764 | 208 (11.8） | 17.8 | 1.64 (1.23-2.21) | 0.001 | 1.62 (1.20-2.20) | 0.002 |
| Participants with elevated nighttime BP | 1610 | 194 (12.0) | 18.2 | 1.50 (1.10-2.06) | 0.011 | 1.42 (1.02-1.98) | 0.037 |
| Participants with no statin use | 1216 | 123 (10.1) | 14.9 | 1.75 (1.18-2.59) | 0.005 | 1.60 (1.06-2.41) | 0.024 |
| **Notes:** full adjusted model: based on minimal sufficient adjustment sets for estimating the total effect of non-dipping pattern on new-onset diebetes: age, gender, drinking status, hypertension duration, baseline prediabetes, BMI, fasting blood glucose, eGFR, serum potassium, serum sodium, mean daytime DBP, ACEI/ARBs use, AHI, nadir SaO_2_, and regular CPAP treatment.  **Abbreviations:** BMI, body mass index; eGFR, estimated glomerular filtration rate; AHI, apnea hypopnea index; SaO_2_, oxygen saturation; ACEI, angiotensin-converting-enzyme inhibitor; ARB, angiotensin II receptor blocker. | | | | | | | |
