# Supplementary material for: Non-dipping blood pressure pattern is associated with higher risk of new-onset diabetes in hypertensive patients with obstructive sleep apnea: UROSAH data
Source: Front Endocrinol (Lausanne). 2023 Feb 16;14:1083179. doi: 10.3389/fendo.2023.1083179 (PMC9978411; doi:10.3389/fendo.2023.1083179)
Supplement: Supplementary file 3 [file Table_3.docx]

| **Supplementary Table 3**. Univariate and multivariate competing risk analysis | | | | |
| --- | --- | --- | --- | --- |
|  | Crude model |  | Full adjusted model |  |
| Variable | Sub-distribution HR (95%CI) | P | Sub-distribution HR (95%CI) | P |
| Dippers | Ref. |  | Ref. |  |
| Non-dippers | 1.73 (1.22, 2.46) | 0.002 | 1.65 (1.14, 2.40) | 0.009 |
| **Notes:** full adjusted model: based on minimal sufficient adjustment sets for estimating the total effect of non-dipping pattern on new-onset diebetes: age, gender, drinking status, hypertension duration, baseline prediabetes, BMI, fasting blood glucose, eGFR, serum potassium, serum sodium, mean daytime DBP, ACEI/ARBs use, AHI, nadir SaO_2_, and regular CPAP treatment. | | | | |
